# Technology mediator: a new role for the reference librarian?

**DOI:** 10.1186/1742-5581-3-10

**Published:** 2006-10-13

**Authors:** David K Howse, Paul J Bracke, Samuel M Keim

**Affiliations:** 1 Arizona Health Sciences Library, 1501 North Campbell Avenue, P.O. Box 245079, Tucson, AZ 85724-5079, USA; 2 Purdue University Libraries, 504 West State Street, West Lafayette, IN 47907-2058, USA; 3 Department of Emergency Medicine, University of Arizona College of Medicine, P.O. Box 245057, Tucson, AZ 85724-5057, USA

## Abstract

The Arizona Health Sciences Library has collaborated with clinical faculty to develop a federated search engine that is useful for meeting real-time clinical information needs. This article proposes a technology mediation role for the reference librarian that was inspired by the project, and describes the collaborative model used for developing technology-mediated services for targeted users.

## Background

The work of the academic health sciences reference librarian is undergoing significant change. Despite decreasing library gate counts [1] and disintermediation caused by the availability of online resources, reference librarians still provide valued guidance to users, who continue to indicate the need for direction in filtering through the world of published medical information [2-4]. Despite the navigational problems they encounter, users expect more immediate and unmediated access to information. By pursuing a technology-mediated layer of service, libraries can offer fine-tuned navigational tools to specific target groups. The authors propose a model whereby the reference librarian acts as a technology mediator, focusing less on the need for clinical knowledge and interpretive skills, which should be left to medical professionals, and more on identifying information resources and improving the presentation of information through technological mediation. The Arizona Health Sciences Library (AHSL) has utilized federated search technology to add such a layer of service for clinical users, and in the process recognized a possible new or revised role for the reference librarian. Vital to this approach is establishing a collaborative, triangular relationship involving reference librarians, information systems professionals, and targeted users.

**Figure 1:**
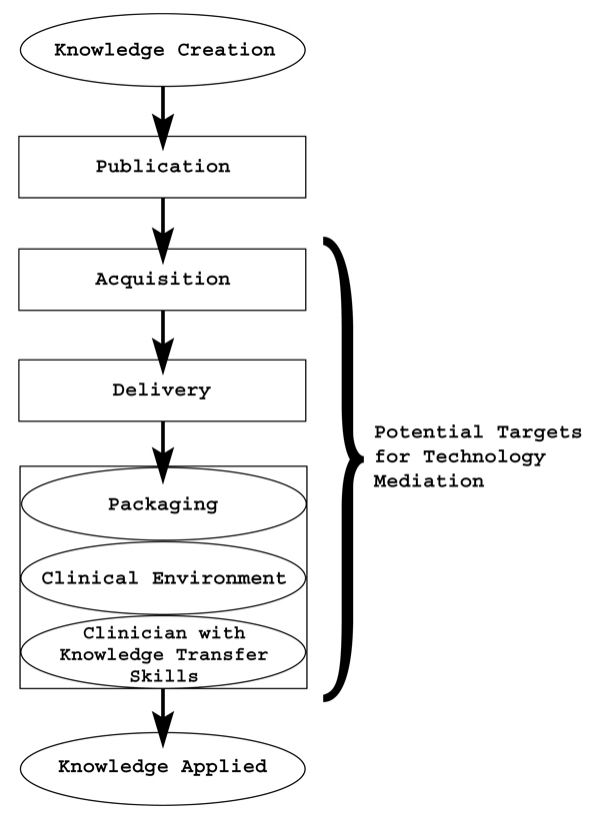
Technology Mediation applied in the clinical setting.

There are several reasons why libraries should pursue better ways to transfer scholarly knowledge to clinicians. Many patients who receive medical care fail to receive the best treatments and might be subjected to harmful therapies and unnecessary tests because clinicians lack pertinent knowledge. High quality evidence supporting most clinical decisions exists in the journal literature, but often does not get translated into consistent decision-making for patients. Evidence-based Medicine, the "the conscientious, explicit, and judicious use of current best evidence in making decisions about the care of individual patients" is an increasingly touted construct for improving clinical care [5-8]. Unfortunately, it has been shown to take up to 20 years for even the most important of these advances to be widely integrated into clinical practice [9]. Many factors are responsible for this dilemma in knowledge transfer (KT), including inadequate continuing education for health professionals and patients; increasingly complex therapies; decreasing resources for health care; and inadequate evidence management [10]. The modern academic health sciences library and medical informatics can help overcome some of these obstacles to knowledge transfer, particularly in the evidence management domain. These tasks include the acquisition and delivery of evidence before the actual translation of knowledge by the clinician at the bedside. (Figure 1) Knowledge transfer or translation may be thought of as the final application of the evidence to the patient's care. Knowledge transfer has been studied from the perspective of trying to understand the issues that determine whether evidence will actually be used by clinicians in practice [11]. One model identifies three main targets to study and focus resources in an attempt to enhance the knowledge translation process: the published knowledge itself, the practice environment around the clinician, and the characteristics of the clinician. (Figure 1) Delivering evidence to the physician in the practice environment is a challenge that can be addressed by first gaining a thorough understanding of the clinical context. Then, by creatively employing technology, it is possible to package knowledge in an appropriate and convenient to use manner. The AHSL found itself most effective in addressing the challenge of packaging information.

One of the most critical obstacles to KT that technological solutions must address is the time constraint that exists in the practice setting [12]. Emerging, frequently updated clinical information tools, such as UpToDate and ACP PIER, as well as content available for Personal Digital Assistants (PDA), are likely popular because of their convenience. Convenient electronic information resources may be reducing the need for librarian-mediated searching. Librarians are consequently challenged to find ways to deliver evidence to the clinical context [13]. One approach has been to train and integrate reference librarians into the clinical environment [14-17]. Similarly, Davidoff and Florance described the need for the informationist, whose ideal skill set would include a combination of clinical knowledge and information science training [18]. The authors presume that many clinicians prefer to control the information-seeking process, and that our efforts should instead be focused on technological mediation.

Creating convenient clinical information tools helps overcome barriers to evidence-based practice, but obstacles to knowledge transfer still remain. For example, although clinicians have shown an increased reliance on Internet-based resources [19,20], a recent review revealed that consultation with colleagues and paper sources remain the first choice when clinical questions arise [21]. Physicians also often mistakenly assume that answers to clinical questions do not exist,[3] further suggesting the need for continued research and innovation into convenient knowledge transfer vehicles and continuing education of clinicians. Shaughnessy et al. likened the "current medical information system" to a "jungle" and provided a map, the "usefulness equation," stating that the usefulness of medical information is inversely related to the work done to obtain it [22]. Connelly et al. demonstrated that "availability and applicability" significantly predict knowledge resource use [23], and Ely et al. noted that "lack of time" was a key obstacle encountered by doctors in their attempts to answer clinical questions [12].

## The Project

Librarians at the Arizona Health Sciences Library (AHSL) have, through observation and focused discussion with users, long-suspected that clinical information-seekers frequently use non-library Internet search engines, even if they have been introduced to the library web site. Navigating the library's web site can be complex for the untrained user, due to the need to select from a myriad of information resources, as well as learning to use multiple product interfaces. Dialogue with Emergency Department (ED) clinicians at our institution suggested they knowingly bypassed excellent resources in favor of simplicity, primarily due to time constraints. The ED, therefore, seemed a sensible clinical setting to assess the knowledge transfer obstacles and seek solutions with impact. We hypothesized that if the library could produce a tool that emulated the simplicity of an Internet search engine, clinicians might be more inclined to use it. As a result, AHSL concentrated its efforts on aggregating and delivering packages of key resource subsets via a search engine.

The project began with meetings between the Emergency Department Residency Program Director and a reference librarian, which focused on the problem of transferring knowledge from the medical literature to physicians in a busy practice environment. Among the possible solutions was the idea of creating a search interface limited to the list of full-text databases and textbooks that would be of immediate use to busy clinicians of that specialty. Needing technical guidance, the Head of the Systems department was consulted. A triangular, collaborative and productive relationship quickly developed between the residency director, the reference librarian and the systems librarian, who brainstormed about the technical feasibility of enhancing access to digital resources for this target group.

The reference librarian coordinated a small number of highly focused meetings during the planning, implementation and troubleshooting stages, which involved all three parties. More frequent meetings took place between the reference librarian and a second party on issues where the third party was not needed. The reference librarian therefore acted as both an advisor and conduit between the systems and clinical parties, in addition to designing the layout of the user interface. The result was a customized federated search tool that was conveniently launched from clinician desktops in their busy emergency department.

The residency director played an important role in two stages of the project – design and promotion. During the design phase, he was instrumental in communicating the clinical decision-making needs of ED clinicians, provided valuable insights into ED workflow, as well as other important contextual information about work within the ED. He also played an important role in promoting the tool to clinicians in the ED, as well as during monthly journal club meetings with residents. The reference librarian promoted use of the search engine during a demonstration at Emergency Grand Rounds, and subsequently visited the ED frequently to both raise awareness of the tool and train users.

The systems librarian role was to translate the input received from the reference librarian and residency director into workable technical specifications. This included a significant amount of dialogue to clarify development priorities and details within the specifications so that rapid prototyping and roll-out could be achieved. He was then involved in working with staff developers to implement the tool. The search engine was locally developed using Cold Fusion and Flash. The tool allows a user to input a keyword search, which is then combined with librarian-constructed search hedges to construct links to pre-executed searches in native interfaces. This allows users a simple interface to powerful search capabilities, including any vocabulary mapping supported within the native interface. Additionally, results are displayed in a hierarchical manner ordered by level of clinical evidence. Currently, the system employs the National Library of Medicine's e-utils service to retrieve the number of hits for PubMed searches, and plans are underway to implement a Z39.50 to Web Services gateway to provide similar functionality for databases that do not support Web Services interfaces. A future article will elaborate further on technical design features, including details about both the user and management interfaces.

The same strategy was used to develop a similar customized search engine for other specialties in the institution. For example, the Pediatrics Residency Director later requested that a customized search tool be developed for that specialty, and a similar process ensued with that group. These web-based federated search engines were launchable from desktops of computers in the respective departments in the immediate vicinity of patient beds. All had customized filters and displayed retrieved references according to an evidence ranking system that addressed the individual needs of these specialties. Site visit statistics, as well as qualitative evidence about the utility and performance of each tool has been favorable. Work is underway to address the needs of new target groups, as well as formally evaluate performance.

## Discussion

While this project introduced a new tool for clinical users, it also fueled ideas about a revised, future role for the reference librarian, as a relationship manager and technology mediator. Of the three parties involved – reference librarian, systems librarian and clinician – the reference librarian possessed the most comprehensive understanding of the operational environments, related to knowledge transfer. Further facilitating the design process was the fact that both the systems and reference librarians are linked by the resources they support. On the other hand, we found that direct dialogue between the clinical and systems parties was much less likely to be productive. By managing this relationship, the reference librarian was able to broker solutions as an intermediary. We found this to be a familiar role and skill set for the reference librarian.

**Figure 2:**
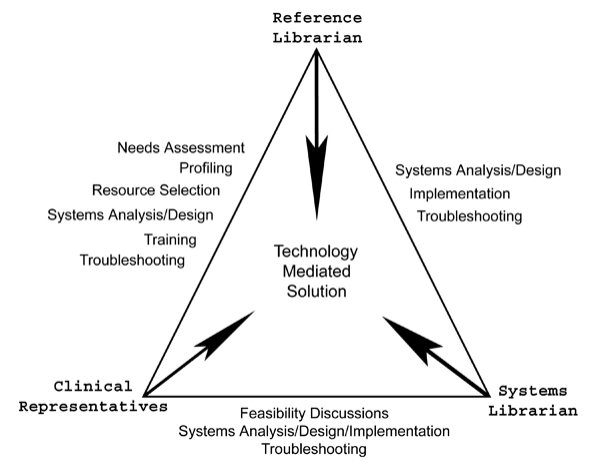
Triangular Communication Model depicting contributions of collaborators and connecting relationships.

Deploying the reference librarian in the role of technology mediator is a logical application of knowledge and skills. By intervening in the user interface, or tool design process, reference librarians can save time and improve navigation for users. The typical reference desk encounter reveals the differences in the interpersonal versus technological approaches. The reference interview consists of the librarian listening to a patron describe an information need, then marrying it to a specific source. The technological intermediary model instead strives to establish convenience in locating information across many potential resources. The first step is for the reference librarian to conduct an information needs assessment, which involves profiling the target user group by gathering knowledge about their 1) environment, 2) typical information seeking behaviors and 3) researching relevant library science and medical literature. In the case of the AHSL, although a set of standard questions was developed to better understand how clinical users typically sought information, the approach to gathering this information was intentionally informal and conversational. The next step is to compile a preliminary list of resources that might be considered valuable by the target group. Then, drawing on expert-level knowledge of database searching, the reference librarian can integrate powerful search strategies into the tool in order to retrieve the subset of content that accommodates the typical, broad needs of the target group. For example, Pediatricians are obviously interested in the subset of evidence-based clinical literature that covers children, and reference librarians can create a search strategy to isolate this subset relatively easily by using age-specific and evidence-based search filters. Once the search strategy for such a subset is created, systems librarians can make the search a permanent part of a web-based search or navigational tool. The accumulated information about the target group can inspire a technology-based service vision. When this vision is communicated effectively to the library's systems professionals, it can result in further investigation of the potential of available technologies to deliver this service vision, and ultimately a powerful and more convenient tool for users. (Figure 2) This triangular communication model illustrates where opportunities for collaboration lie between the participants. The reference librarian mediates at each discussion or key project-related situation.

The technology mediation approach differs from the clinical librarian or informationist model by removing the responsibility for searching and critical appraisal. According to Davidoff and Florance's description, the informationist, most likely working in the clinical setting, must be able to critically appraise and interpret the medical literature, then facilitate the transfer of this knowledge to the clinician treating the patient [18]. Unfortunately, critical appraisal is often challenging for the reference librarian without foundational clinical exposure, and biostatistics and research design training. Furthermore, this model presumes that clinicians would be consistently willing to communicate information needs to such a professional, and relinquish control of the entire information seeking exercise. While there are merits and drawbacks to both approaches, it is likely that many clinicians would prefer to remain in control of the process, especially if convenience continues to improve with technological innovation.

While identifying opportunities to improve convenience principally begins with the reference librarian, users must be active participants in the process. Users contribute fundamental knowledge about the context of the information need, obstacles influencing one's ability to find answers, and later, feedback about the success or failure of tested solutions. In order to involve clinical users, it is crucial to establish trust, gained simply by convincing them that the goal is to improve their work lives. For example, promoting use of a specific information resource that has impressive, powerful search capabilities, does not guarantee users will embrace it, especially if it takes considerable time to master. Conversely, it has been shown that social influence achieves more success when the goal is to convince users to adopt technology [24]. Involving the users is crucial to success, because they advocate their own solutions. The relationship depicted here employed an approach that has best been described as relationship management (RM). A business concept described by Parvatiya and Sheth, RM refers to a process of acquiring, retaining and partnering with selective customers to create superior value for the company and the customer [25]. As noted by Enyeart and Weaver, this approach can be applied to the health sciences librarianship as a means of driving the development of services [26]. In order to meet the needs of selective customers – clinicians – the AHSL added an additional layer of superior value – a customized search engine that was largely designed to the users' specifications. The result is a tool that permits independent searching of content specified by the users. Similar examples in the online business world reveal how technology has changed how businesses interface with customers, leading to less interpersonal mediation, and customers have embraced this independence. Online banking and direct sales of books are examples of how online business portals have eliminated middlemen to allow producers to interact directly with their customers. Library websites now provide patrons with direct access to databases and services, and library patrons are similarly willing to renew books, and perform catalog and literature searches. The difference for libraries is that, functioning as a resource aggregator, they must include multiple vendor interfaces into their web sites, and users are faced with the resulting navigational inconveniences. The reference librarian can be a driving force behind solving these navigational problems.

## Conclusion

As database aggregators, libraries are in the unique position of being able to create a navigational layer that can select and connect multiple resources logically for users, who increasingly demand convenience and simplicity. The first obstacle to overcome is establishing a connection between the three parties with critical knowledge bases – systems, resources, and users. Identifying and enlisting these parties presents a challenge, especially for smaller organizations with limited resources. In the world of academic health sciences libraries, reference librarians are best suited to initiating and maintaining this relationship, as well as advocating for navigational tools that create added value and convenience for users. By partnering with library systems experts and clinical users, creative solutions can be brokered that could have a positive impact both on educating clinicians, and ultimately patient care.
